# Ultrafast carrier dynamics in Ge by ultra-broadband mid-infrared probe spectroscopy

**DOI:** 10.1038/srep40492

**Published:** 2017-01-11

**Authors:** Tien-Tien Yeh, Hideto Shirai, Chien-Ming Tu, Takao Fuji, Takayoshi Kobayashi, Chih-Wei Luo

**Affiliations:** 1 Department of Electrophysics, National Chiao Tung University, Hsinchu 300, Taiwan; 2 Institute for Molecular Science, 38 Nishigonaka, Myodaiji, Okazaki 444-8585, Japan; 3 Advanced Ultrafast Laser Research Center, and Department of Engineering Science, Faculty of Informatics and Engineering, University of Electro-Communications, 1-5 1 Chofugaoka, Chofu, Tokyo 182-8585, Japan

## Abstract

In this study, we carried out 800-nm pump and ultra-broadband mid-infrared (MIR) probe spectroscopy with high time-resolution (70 fs) in bulk Ge. By fitting the time-resolved difference reflection spectra [Δ*R(ω*)/*R(ω*)] with the Drude model in the 200–5000 cm^−1^ region, the time-dependent plasma frequency and scattering rate have been obtained. Through the calculation, we can further get the time-dependent photoexcited carrier concentration and carrier mobility. The Auger recombination essentially dominates the fast relaxation of photoexcited carriers within 100 ps followed by slow relaxation due to diffusion. Additionally, a novel oscillation feature is clearly found in time-resolved difference reflection spectra around 2000 cm^−1^ especially for high pump fluence, which is the Lorentz oscillation lasting for about 20 ps due to the Coulomb force exerted just after the excitation.

For semiconductors, the physical parameters, e.g., carrier scattering rate, mobility and concentration, are important for applications in electronics and opt-electronics, especially for high-speed devices such as photodetectors. The infrared (IR) absorption spectroscopy has demonstrated to be a convincing method for investigating the optical properties of materials in the IR region and some other physical parameters explicitly relevant to the IR spectra^1^. Generally, the whole absorption feature in common materials typically extends rather broad spectral range. Consequently, the broadband spectrum can capture the absorption feature even without studying the dependence of carrier concentration or effective mass. However, the conventional infrared (IR) absorption spectroscopy can provide only stationary information without dynamic behavior. More than a decade ago, by the intensity modulation of IR light source, the nanosecond (ns) time resolution was achieved^2^. Higher time resolution experiment has been desired. Recently, Fuji *et al*.^3^,^4^,^5^ generated sub-10 fs ultra-broadband IR pulses in air plasma with much broader width over 5000 cm^−1^ than that generated by different frequency generation (DFG) in several nonlinear crystals^6^,^7^. By utilizing such pulsed source in the optical pump-probe experiments, it can immediately provide the time-dependent physical parameters for dynamic investigations and applications.

Based on this novel ultrafast light source, Shirai *et al*.^8^ performed the transient pump-probe spectroscopy for Ge bulk crystal with 70-fs-time-resolution. However, the transient spectra in Ge obtained by the optical pump mid-IR probe spectroscopy have not been discussed in detail yet. In this paper, we present more analyses and discussions for the difference reflection spectra [Δ*R(ω*)/*R(ω*)] in Ge because of the difficulty in transmission spectra due to the opaque property in the range below 2 μm. Besides, the difference transmission signal (Δ*T*/*T*) is heavily suppressed by the absorption of surface excited carriers to cause the difficulties for the analyses. Comparing with the transmission configuration for the practical applications, the measurements of Δ*R(ω*)/*R(ω*) in the reflection configuration are more widely applied to various types of materials, including opaque materials, transparent materials^9^, bulk^6^,^10^, thin films^11^, and hetero-structures^12^,^13^. Moreover, the ultra-broadband and 70-fs time-resolved spectra developed in this study are wide enough to provide more reliable fitting results and able to fully reveal the evolution of most of the features in spectra. For example, we have obtained the time-dependent carrier plasma frequency, concentration, scattering rate, and mobility by using the free carrier absorption model. Additionally, we discuss the mechanism of photoexcited carrier relaxation processes through the numerical analyses. Last but not least, we have found that a novel oscillation feature in time-resolved difference reflection spectra around 2000 cm^−1^ prominently appearing in the case of high pumping fluence, which is concluded to be due to the Lorentz oscillation with the Coulomb force within 20 ps.

## Experiments

**Figure 1:**
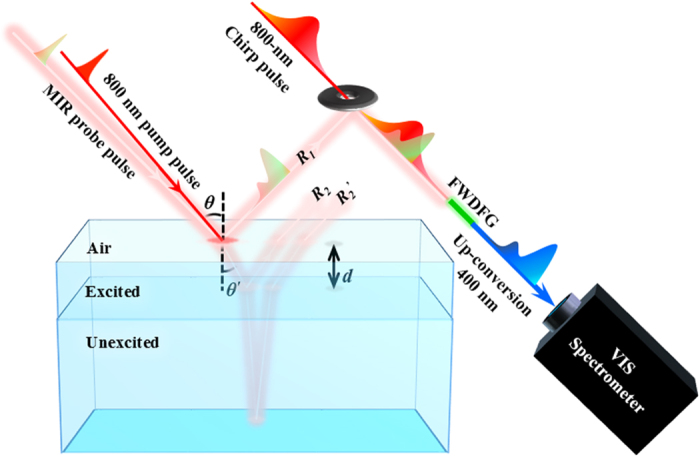
Schematics of the 800-nm pump and ultra-broadband mid-infrared (MIR) probe spectroscopy and the detection scheme with chirped-pulse upconversion. R_1_: the 1^st^ reflection of probe beam. R_2_: the 2^nd^ reflection of probe beam from the interface between excited and unexcited regions. R_2_’: the 2^nd^ reflection of probe beam from the backside of a Ge sample. *d*: the depth of excited region. FWDFG: four-wave difference frequency generation. *θ*: incident angle of probe beam. *θ*′: refraction angle of probe beam.

An intrinsic (100) Ge crystal wafer of 0.5-mm thick was used as a sample. We use a Ti:sapphire multipass amplifier system (800 nm, 30 fs, 0.85 mJ at 1 KHz, Femtopower compactPro, FEMTOLASERS) as a light source. The output pulse is split into three with two beam splitters. The first pulse is used to generate an ultra-broadband mid-infrared (MIR) probe pulse, the second pulse is used as an optical pump pulse, and the third pulse is used for a chirped pulse. The MIR probe pulse (*ω*_*0*_) with 8.2-fs-pulse duration is generated by combining the fundamental (800 nm, *ω*_*1*_) and second harmonic (SH, 400 nm, *ω*_*2*_) pulses with the four-wave difference frequency generation (FWDFG, *ω*_*1*_ + *ω*_*1*_ − *ω*_*2*_ → *ω*_*0*_) through filamentation in air. By using the optical pump (800 nm) and ultra-broadband MIR probe spectroscopy, the reflectivity change (Δ*R*/*R*) transients of Ge in the region from 200 to 5000 cm^−1^ can be obtained. For detection as shown in Fig. 1, the MIR pulses reflected from the sample are converted to visible pulses (*ω*_*2*_, 400–500 nm) for detection through chirped-pulse up conversion (CPU, *ω*_*1*_ + *ω*_*1*_ − *ω*_*0*_ → *ω*_*2*_). The chirped pulse is obtained from the 800 nm pulse through four BK7 (*t* = 10 mm) substrates and a ZnSe (*t* = 5 mm) substrate at the Brewster angles. The up-converted spectrum is measured by electron-multiplying charge-coupled device camera (EMCCD, SP-2358 and ProEM + 1600, Princeton Instruments). The Δ*R*/*R* spectrum is obtained by up-converted probe beam spectrum for each delay with or without pump. To prevent the absorption of carbon dioxide and water vapor, the whole system is purged with nitrogen. The details of experiments have been reported in our previous work^8^.

## Results and Discussion

### Photoexcited carrier dynamics

**Figure 2:**
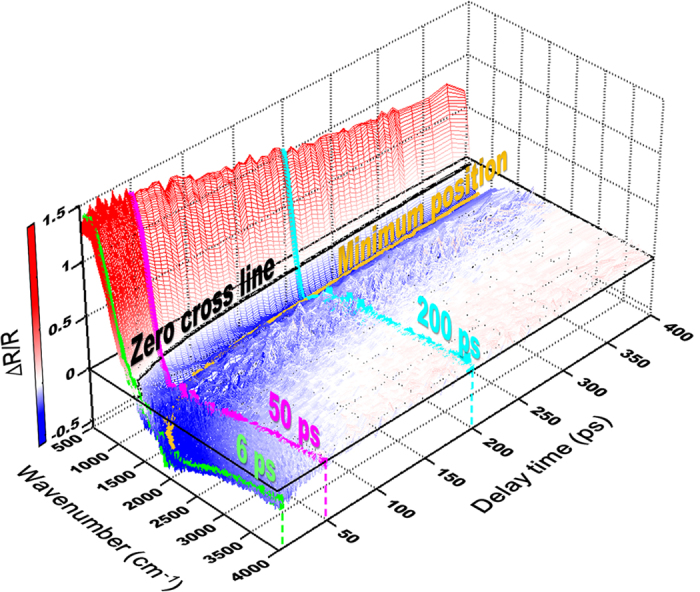
The reflectivity change (Δ*R*/*R*) transients as a function of wavenumbers in Ge after exciting with the pump fluence of 135 μJ/cm^2^.

**Figure 3:**
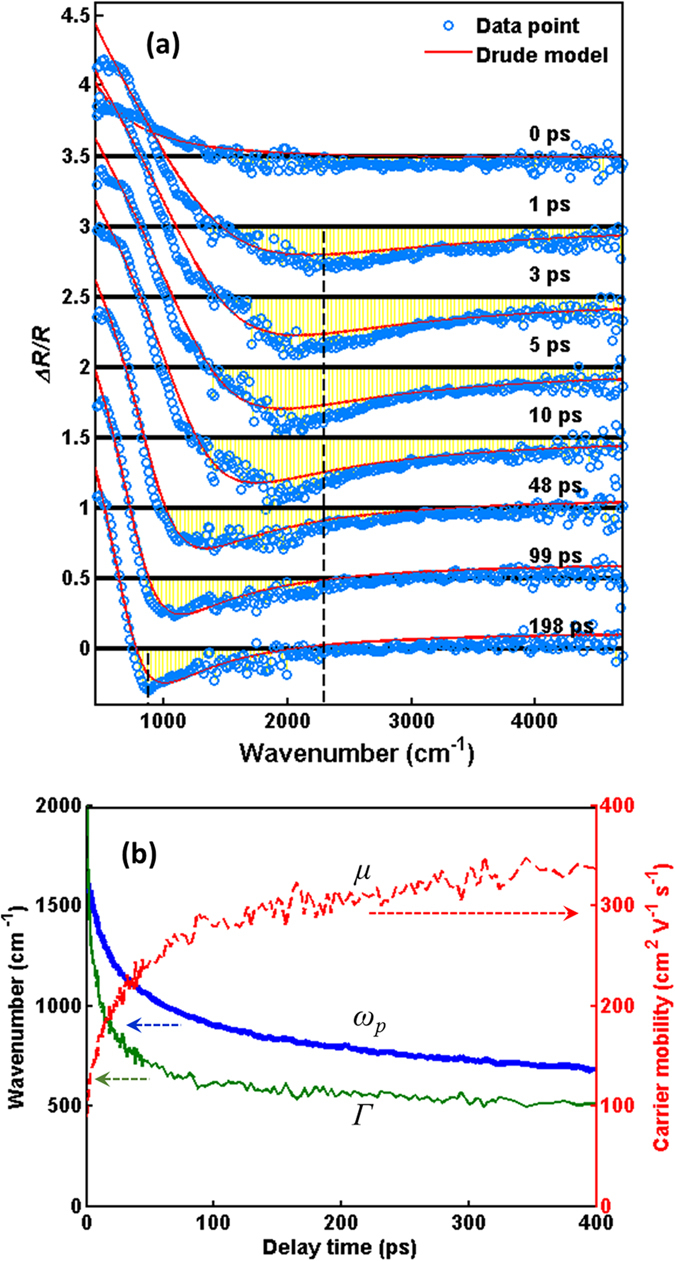
(**a**) The time-dependent Δ*R*/*R* as a function of wavenumbers in Ge at different delay time, which obtained from Fig. 2. The blue-opened circles are experimental data. The red-dashed lines are the fitting curves with the Drude model of Eq. (1). (**b**) The time evolution of carrier mobility (*μ*), plasma frequency (*ω*_*p*_), and scattering rate (*Γ*) obtained from the fitting in (**a**).

Figure 2 shows an example of Δ*R*/*R* spectrum of Ge in MIR region. The feature of plasma edge with positive Δ*R*/*R* (red color) below 1000 cm^−1^ and negative Δ*R*/*R* (blue color) above 1000 cm^−1^ can be clearly observed from zero delay time up to 400 ps. As shown in Fig. 3(a), the Δ*R*/*R* dramatically shrinks with increasing wavenumber and it crosses zero to negative in the range of 750–2000 cm^−1^. Additionally, the position of minimum Δ*R*/*R* (or plasma edge) gradually shifts toward a low-wavenumber region as the delay time increases; meanwhile, the negative hump (yellow area) also gradually narrow down. Similar phenomena were observed also by Carroll *et al*.^14^ in bulk Ge with 100-ps resolution. These features can be qualitatively described by the Drude model, which treats the free carriers in a solid as the point charges with random collisions. Using the stationary reflectance *R* = 0.24 for Ge^8^, the dynamic reflectance *R(t*) = *R* + Δ*R(t*) = *R* × {1 + [Δ*R(t*)/*R*]} is used to fit the experimental data in Fig. 2. By using the software of RefFIT^15^, the *R(t*) of p-wave IR probe can be fitted with [see the red lines in Fig. 3(a)]


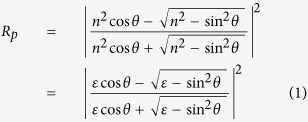


where *n*: complex refractive index, *θ* = 45°: incident angle, and *ε(ω*) complex dielectric constant given by


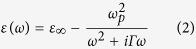


(*ω*: angular frequency, *ω*_*p*_: plasma frequency, *Γ*: scattering rate, and *ε*_∞_: permittivity at infinite frequency). Experimental incident angle is set at *θ* = 45°. For the best fit, the fitting parameter of *ε*_∞_ is between 15 and 18, which closes to the theoretical estimation of *ε*_∞_ = 16^16^. Besides, the time evolution of *ω*_*p*_ and *Γ* can be obtained as in Fig. 3(b). Both *ω*_*p*_ and *Γ* significantly decrease with increasing the delay time and then remain constant for the longer delay time. On the contrary, the carrier mobility *μ* (=*eτ*/m*, where is the electron charge, m* is the carrier effective mass, *τ* is the average scattering time, which is equal to 1/*Γ*) rises with increasing the delay time due to the reduction of carrier concentration^17^,^18^,^19^. After 200 ps, the carrier mobility *μ* maintains to be ~350 cm^2^ V^−1^ s^−1^.

Moreover, the photoexcited carriers are only generated near the surface of Ge sample due to the small penetration depth *l*_*800*_ of 0.2 μm for 800-nm pump beam (defined as the inverse of absorption coefficient *α*, where *α* = 49322.85 cm^−1^ at 800 nm^20^). For the ultra-broadband MIR probe beam, the penetration depth is wavelength- and time-dependent. According to *l*_*MIR*_(*t*) = *c*/[2*n*_2_(*t)ω*], where *c* is the vacuum light speed, *n*_*2*_(*t*) is the imaginary part of time-dependent refractive index and *ω* is the MIR angular frequency, the penetration depth *l*_*MIR*_(*t*) of MIR probe beam is estimated at different delay time. Prior to the pump pulse excitation, Ge is partially transparent (~30%) in the MIR range. The penetration depth of MIR probe beam is only a few μm at 3 ps after pump pulse excitation. This is much smaller than the sample thickness 500 μm. However, after 200 ps, it becomes larger than the sample thickness reaching to a few hundred μm resulting in the appearance of backside reflection feature (*R*’_2_ in Fig. 1) of the sample. Additionally, the detection depth *l*_*d*_ of CPU system can be estimated by


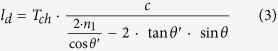


where *T*_ch_ = 400 fs is the duration of chirped pulse, *θ*′ is the refraction angle in Ge (see Fig. 1), *n*_*1*_ is the real part of refractive index of Ge. Taking *n*_*1*_ = 4^21^, *θ* = 45°, and *θ*′ = 10.2°, the detecting depth *l*_*d*_ is 15.2 μm, which is much smaller than the sample thickness. Therefore, our measurements are free from signal contamination by the backside reflection in the sample. However, *l*_*d*_ = 15.2 μm is longer than the 0.2-μm penetration depth of pump beam. Therefore, interesting to say that the probe MIR beam can monitor both excited and unexcited regions simultaneously under the present study condition. The detailed analyses of the carrier relaxation processes is discussed in the following sections.

### Transient carrier diffusion effect

**Figure 4:**
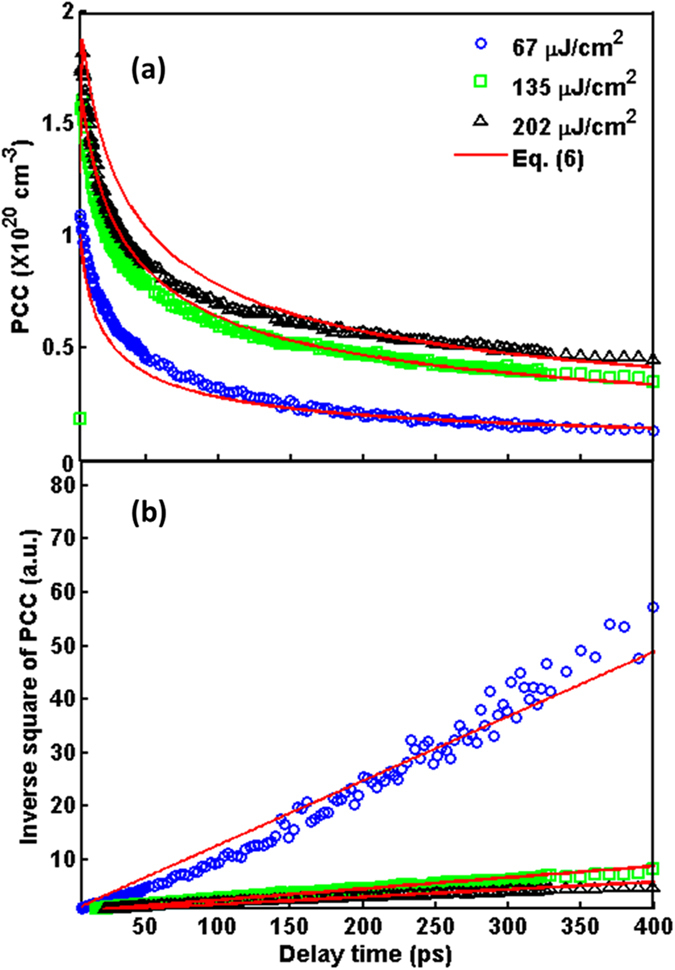
(**a**) Time evolution of photoexcited carrier concentration (PCC) obtained from Fig. 3(b) with Eq. (4) at various pump fluences. The red-solid lines show the fitting with Eq. (6). (**b**) Inverse square of photoexcited carrier concentration (PCC) as a function of delay time. The red-solid lines show the fitting of Eq. (6) with x~0.

As mentioned in the last section, the photoexcited carriers are generated nearby the surface of Ge (within 0.2 μm) by pump beam. Besides the short-range collisions among photoexcited carriers, the photoexcited carriers also diffuse from the excited region to the unexcited part due to the spatial gradient of photoexcited carrier concentration. The time evolution of carrier concentration *N* can be obtained by Eq. (4) 22


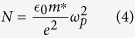


where m* denotes the carrier effective mass, *ε*_*0*_ is the vacuum permittivity, *e* is the electron charge. The effective mass is 0.34*m*_*e*_ for a split-off hole^23^, where *m*_*e*_ is the electron mass. Because of the time-dependent *ω*_*p*_ as shown in Fig. 3(b), we can further obtain the time evolution of carrier concentration *N*, which decreases gradually after pumping [see Fig. 4(a)]. Moreover, the decrease of carrier concentration further causes the red-shift of the position of spectrum minimum.

By changing the pump fluence (*F*) from 67 to 135 μJ/cm^2^, the photoexcited carrier concentration increases significantly. However, the photoexcited carrier concentration shows saturation when the pump fluence is further increased from 135 to 202 μJ/cm^2^. To explain the reduction of photoexcited carrier concentration quantitatively, the following differential equation with diffusion term is invoked^24^,


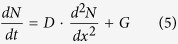


where *D* is the diffusion coefficient, *G* is the carrier generation rate assuming to be much faster than the diffusion rate. By solving Eq. (5), we can obtain the analytic solution as follows,


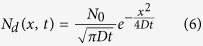


where *N*_*0*_ is the total number of photoexcited carriers, which can be determined by integrating *N*_*d*_ along 1-dimension depth direction *x* perpendicular to the sample surface. In the range closed to surface of Ge (i.e. *x* ~ 0), 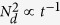 is expected in case the dynamics is only due to the diffusion process. By fitting the data in Fig. 4(b) via Eq. (6) with x~0, the diffusion coefficient can be obtained from the slope, e.g. *D* = 66 cm^2^/s for the pump fluence of 67 μJ/cm^2^, which is consistent with the theoretical calculation of 65 cm^2^/s^25^. Moreover, the diffusion coefficient *D* significantly decreases to 20 and 18 cm^2^/s when the pump fluence increases to 135 and 202 μJ/cm^2^, respectively, as listed in Table 1.

### High-order transient effects

Even though the diffusion model qualitatively reproduces the dynamics of photoexcited carrier concentration especially in the long delay-time range, the difference between experimental data and diffusion model is substantial at shorter delay than 150 ps as shown in Figs 4(a) and 4(b). This implies that other mechanisms might involve in the relaxation processes of photoexcited carriers of Ge in short delay time, such as bandgap renormalization, recombination effect, and intervalley scattering. The bandgap renormalization usually happens after short pulse excitation because of intimate relation between the gap-size and the carrier concentration. However, to observe the bandgap renormalization effect, the measurements of transmittance^26^,^27^ or photoluminescence^28^ are indispensable. In Hamberg’s works^26^, moreover, they propose a clear picture for the roles of reflectance and transmittance, which can provide the information of plasma oscillation and the band absorption, respectively. Therefore, the difference reflection spectra in this study would primarily represent the signals of plasma oscillation rather than the bandgap renormalization, which can be further more definitively neglected in our fittings.

**Figure 5:**
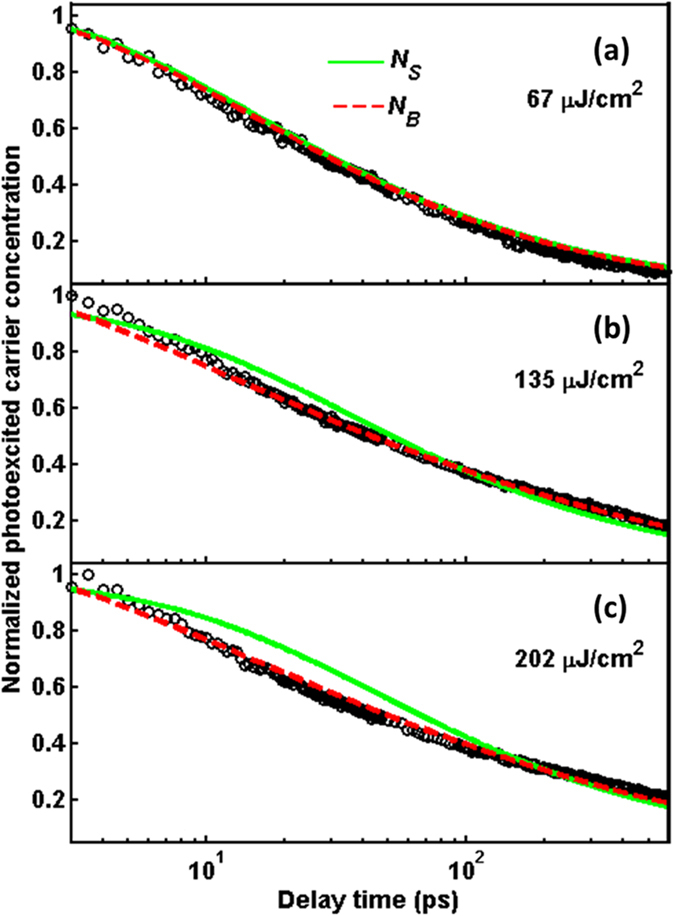
The experimental data in Fig. 4(a) are presented in a normalized semi-logarithmic scale at various pump fluences of (**a**) 67 μJ/cm^2^, (**b**) 135 μJ/cm^2^, (**c**) 202 μJ/cm^2^. The green-solid and red-dashed lines are fitted by the Eqs. (8) and (9), respectively.

Ge is an indirect-bandgap semiconductor material. After photoexcitation, the intervalley scattering from the Γ valley to a side valley dominates the carrier transformation in hundreds of fs^29^,^30^, and take few μs for recombination at the Γ point^31^,^32^. This is the main process for changing the photoexcited carrier concentration in Ge. Especially for the pump in p-type Ge, the relaxation processes from split-off hole band to upper hole band and scattering between heavy hole and light hole bands could be observed^33^,^34^. However, the carrier relaxation processes inside of the split-off band, heavy-hole band, and light hole band do not induce the changes of photoexcited carrier concentration. Actually, we do observe the reduction of photoexcited carrier concentration in short delay time region, which cannot be simply explained by the diffusion mechanism. Therefore, several other relaxation processes, e.g. the recombination, surface recombination, radiative recombination, and Auger process^35^, should be involved in analysis particularly for high pump fluence as in the following equation,


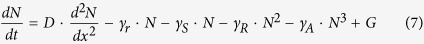


where *N* is the carrier concentration, *D* is the diffusion coefficient, *γ*_*r*_ is the recombination rate, *γ*_*S*_ is the surface recombination coefficient, *γ*_*R*_ is the radiative recombination coefficient, *γ*_*A*_ is the Auger coefficient, and *G* is the Gaussian-type generation function for a laser pulse. In order to solve the nonlinear Eq. (7), it is rewritten by the Crank-Nicolson form^36^ as described in Supplementary Information. If we simply consider that the photoexcited carriers are just generated or only can be detected on the surface, the photoexcited carrier concentration on surface can be expressed as


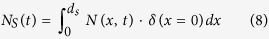


where *N(x, t*) is the solution of Eq. (7), *d*_*s*_ is the sample thickness and *δ(x* = 0) is the Dirac delta function. As shown by the green lines in Fig. 5, the experimental data are fitted well with the Eq. (8) for the case of low pump fluence 67 μJ/cm^2^. However, it cannot be applied to the cases of high pump fluence, especially below 100 ps.

Additionally, the penetration depth *l*_*800*_ of 800-nm pump beam is about 0.2 μm. As mentioned above, the detection depth *l*_*d*_ is around 15.2 μm. This indicates that it is necessary to consider all photoexcited carriers in bulk rather than only on the surface. Therefore, the photoexcited carrier concentration in bulk is expressed as


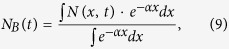


where *N(x, t*) is the solution of Eq. (7) and *α* is the absorption coefficient (=1/*l*_*MIR*_). The experimental data in Fig. 5 can fit well with Eq. (9) for different pump fluence. When pump fluence increases from 65 to 135 μJ/cm^2^, the substantial decrease in *α* is clearly shown in Table 1 indicating that a longer penetration depth (*l*_*MIR*_ ~ 0.8 μm) of MIR probe beam for higher pump fluence. Moreover, for the same *l*_*MIR*_, the saturation effect is also found for further increase in the pump fluence to 202 μJ/cm^2^.

Based on the well-fit red-dashed lines in Fig. 5, we can further discuss the importance of each term in Eq. (7). For the second term of Eq. (7), the time scale of recombination is in the order of μs, which is much longer than the measuring range of 400 ps in this study. In the third term of Eq. (7), the surface band bending causes the surface recombination. Without the special surface treatment, the surface recombination velocity is about 1300 cm/s^37^, and its time scale is still in μs. For the radiative recombination [the fourth term of Eq. (7)], the recombination rate in the bulk Ge with indirect band gap is ~10^−10^ cm^3^/s^38^, which is smaller than the commonly found value of the order of 10^−8^ cm^3^/s for direct band gap. Thus, the relaxation process of radiative recombination is also negligible in the present experimental condition (the critical value of *γ*_*R*_ for this study is 10^−9^ cm^3^/s).

As discussed above, the Auger effect dominates the relaxation within 100 ps. The fitting results in Table 1 show that the Auger coefficient *γ*_*A*_ (2–3 × 10^−30^ cm^6^/s) is independent of pump fluence (*F*), i.e. the photoexcited carrier concentration (*N*). According to the relation of 1/*τ*_*A*_ = *γ*_*A*_ *·* *N*^*2* ^38, we further estimate the recombination time *τ*_*A*_ of Auger process, which is in the range of 13–30 ps and dependent on pump fluence. For high pump fluence, e.g. *F* = 135 and 202 μJ/cm^2^, the *τ*_*A*_ becomes small to imply that the efficiency of Auger process would be dramatically enhanced by high photoexcited carrier concentration. On the other hand, the diffusion coefficient decreases down to 20 cm^2^/s with including the Auger process. By the Einstein relation, the value of *D*/*μ* at high carrier concentration is ~0.07^39^. Taking *μ* = 350 cm^2^ V^−1^ s^−1^ obtained in Fig. 3, thus, the *D* becomes 24.5 cm^2^/s which is consistent with the fitting results listed in Table 1.

### Lorentz force for the photoexcited carriers

**Figure 6:**
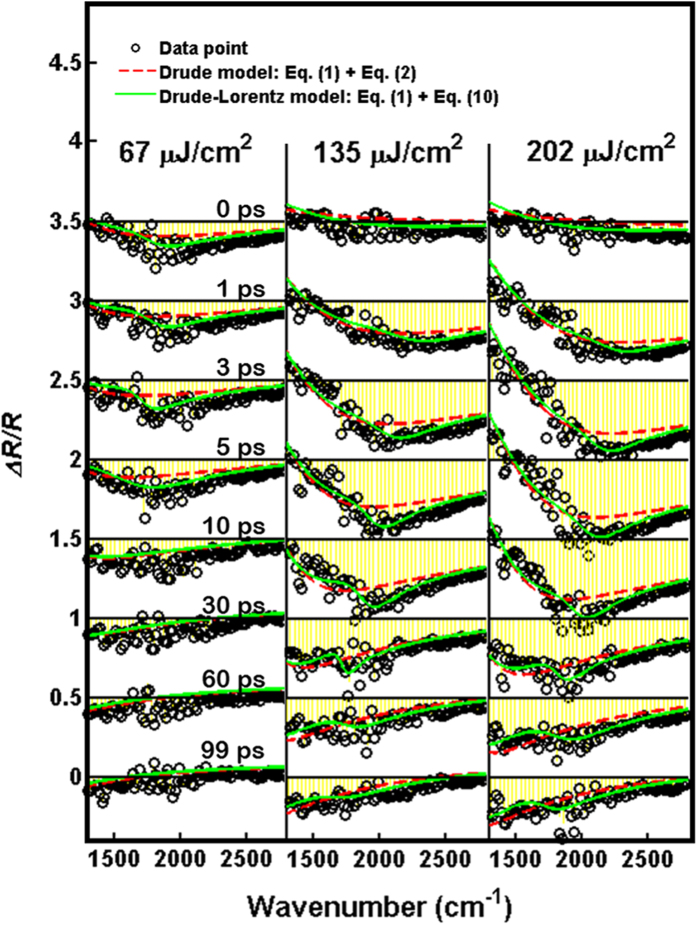
The time-dependent Δ*R*/*R* as a function of wavenumbers in Ge at different delay time, which obtained from Fig. 2 **at various pump fluences.** The origin of abscissa is shifted by 0.5 for each column from the bottom to the top. The black-opened circles are experimental data. The red-dashed lines are the fitting curves with the Drude model of Eqs. (1) and (2). The green-solid lines are the fitting curves with the Drude-Lorentz model of Eqs. (1) and (10).

A closer look at the wavenumber dependence of Δ*R*/*R* at several delay times in Fig. 6 reveals the fitting of Drude model suffers a significant deviation around 2000 cm^−1^, especially for high pump fluence. This implies that some driving forces exist among the photoexcited carriers, which we ascribe to the Lorentz force. Here, we further modified the Drude model with including the Lorentz force, i.e. the so-called Drude-Lorentz model^40^. In Eq. (1), thus, the angular frequency-dependent permittivity is given by


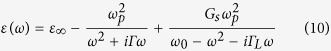


where *ε*_∞_ is the permittivity at an infinite frequency, *ω* is the frequency, *ω*_*p*_ is the plasma frequency, *Γ* is the scattering rate, *G*_*s*_ is related to the oscillator strengths, *ω*_*0*_ is the resonance frequency, and *Γ*_*L*_ is the damping coefficient.

**Figure 7:**
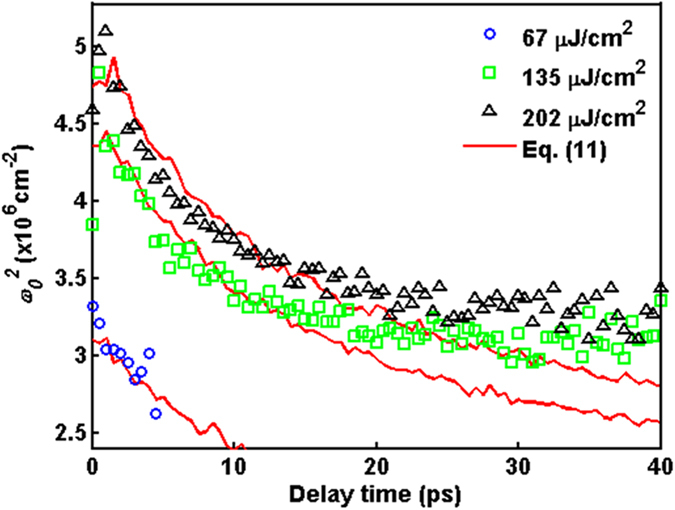
The square of resonance frequency 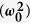 of Lorentz term in Eq. (10) as a function of delay time for various pump fluences. The red-solid lines are obtained using Eq. (11).

As shown in Fig. 6, the green-solid lines of Drude-Lorentz model can fit the Δ*R*/*R* rather well at different delay time. Interestingly, the difference between Drude model and Drude-Lorentz model, i.e. the Lorentz term, is strongly dependent on the pump fluence and delay time. In the cases of high pump fluence, the Lorentz term becomes more dominate and survives for longer time. From the fitting in Fig. 6, the time-dependent resonance frequency *ω*_*0*_ can be obtained as shown in Fig. 7. For all pump fluence, *ω*_*0*_ shows the remarkable red shift below 20 ps.

These results indicate that the photoexcited carriers are bound by a kind of spring force *F*_*s*_ = m*ω_*0*_^*2*^*r* with distance *r*. If the Coulomb collision could serve as the spring force, the carrier would be pulled back by the Coulomb force. Even though the paths and directions of collision are random, the motion of carriers can be considered as a simple harmonic oscillation along a specific direction within short delay time. Here, we simply adopted the Coulomb force *F*_*C*_ to be the spring force *F*_*s*_, which is just the binding force in Lorentz term. Thus, we have *F*_*C*_ = *F*_*s*_ + *c* (where *c* is a phenomenological proportionality constant), and then the *ω*_*0*_ can be expressed as


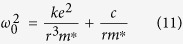


where *k* is the Coulomb’s constant, *r* is the effective distance between the neighboring carriers (which is estimated by 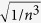, and *n* is the time-dependent carrier concentration), m* is the effective mass, and *c* is 6 × 10^−11^ N. As shown in Fig. 7, Eq. (11) can fit the resonance frequency *ω*_*0*_ quite well below 20 ps. These results indicate that the oscillating feature of Δ*R*/*R* around 2000 cm^−1^ come from the Lorentz oscillation. Moreover, this Lorentz oscillation is driven by the Coulomb force during the collision among the photoexcited carriers.

## Summary

We have studied the photoexcited carrier dynamics in Ge using 800-nm pump and ultra-broadband MIR probe spectroscopy. The time evolutions of carrier mobility, plasma frequency, scattering rate, and carrier concentration have been extracted through the wavelength- (from 200 to 5000 cm^−1^) and time-dependent (below 400 ps) Δ*R*/*R* by fitting with the Drude model. For the reduction of photoexcited carrier concentration, the Auger recombination with the Auger coefficient of 2–3 × 10^−30^ cm^6^/s dominates the relaxation processes of photoexcited carriers within 100 ps. On the other hand, the long-timescale relaxation process is dominated by the diffusion effect with diffusion coefficient of about 20 cm^2^/s. Moreover, a novel oscillation feature is clearly observed in time-dependent trace of Δ*R*/*R* around 2000 cm^−1^ especially in the cases of high pump fluence, which is considered to be due to the Lorentz oscillation raised by the Coulomb force exerted just after excitation.

## Additional Information

**How to cite this article**: Yeh, T.-T. *et al*. Ultrafast carrier dynamics in Ge by ultra-broadband mid-infrared probe spectroscopy. *Sci. Rep.* **7**, 40492; doi: 10.1038/srep40492 (2017).

**Publisher's note:** Springer Nature remains neutral with regard to jurisdictional claims in published maps and institutional affiliations.

## Supplementary Material

Supplementary Information

## Figures and Tables

**Table 1:** List of fitting parameters in Fig. 4, Fig. 5 and previous works.

Type of PCC	*F* (μJ/cm^2^)	Experimental method	*N* (×10^20^ 1/cm^3^)	*D* (cm^2^/s)	*γ*_*A*_ (×10^−30^ cm^6^/s)	*τ*_*A*_ (ps)	*α* (×10^−5^ 1/cm)
*N*_*d*_	67	Current work	1.3	66	—	—	—
	135		1.6	20	—	—	—
	202		1.8	18	—	—	—
*N*_*S*_	67	Current work	1.3	20	1.2	49.3	—
	135		1.6	8	0.5	78.1	—
	202		1.8	8	0.5	61.7	—
*N*_*B*_	67	Current work	1.3	20	2.0	29.6	1/1.2
	135		1.6	20	3.0	13.0	1/8
	202		1.8	20	2.0	15.4	1/8
—	—	Calculation^25^		65	—	—	—
—	—	1.06-μm pump MIR probe^38^	0.7	—	3.2	31.9	—
—	7400	1.06-μm pump 1.55-μm probe^41^	3.4	—	0.11	78.6	—
—	—	transient gratings^42^	0.17	53	—	—	—

Type of PCC: type of photoexcited carrier concentration. *N*: photoexcited carrier concentration. *N*_*d*_: photoexcited carrier concentration with diffusion effect [via Eq. (6)]. *N*_*S*_: photoexcited carrier concentration on surface [via Eq. (8)]. *N*_*B*_: photoexcited carrier concentration in bulk [via Eq. (9)]. *F*: pump fluence. *D*: diffusion coefficient. *γ*_*A*_: Auger coefficient. *τ*_*A*_ = 1/(*γ*_*A*_ · *N*^*2*^). *α*: absorption coefficient.
