# Supplementary material for: Ultrafast carrier dynamics in Ge by ultra-broadband mid-infrared probe spectroscopy
Source: Sci Rep. 2017 Jan 11;7:40492. doi: 10.1038/srep40492 (PMC5225453; doi:10.1038/srep40492)
Supplement: Supplementary Information [file srep40492-s1.pdf]

Supplementary Information

Ultrafast carrier dynamics in Ge by ultra-broadband mid-infrared probe spectroscopy

Tien-Tien Yeh[1], Hideto Shirai[2], Chien Ming Tu[1], Takao Fuji[2], Takayoshi Kobayashi[1,3], and Chih-Wei Luo[1,*]

[1]Department of Electrophysics, National Chiao Tung University, Hsinchu 300, Taiwan

[2]Institute for Molecular Science, 38 Nishigonaka, Myodaiji, Okazaki 444-8585, Japan

[3]Advanced Ultrafast Laser Research Center, and Department of Engineering Science, Faculty of Informatics and Engineering, University of Electro-Communications, 1-5 1 Chofugaoka, Chofu, Tokyo 182-8585, Japan

* Address correspondence to:

Prof. Chih-Wei Luo, Department of Electrophysics, National Chiao Tung University, Hsinchu 300, Taiwan

Email: cwluo@mail.nctu.edu.tw

I. Crank-Nicolson form

The Crank-Nicolson form[1] of Eq. (7) is

$$\frac{N_{\mathrm{i}}^{j+1} - N_{\mathrm{i}}^{j}}{\Delta t} = D\,\frac{N_{i+1}^{j+1} - 2N_{i}^{j+1} + N_{i-1}^{j+1} + N_{i+1}^{j} - 2N_{i}^{j} + N_{i-1}^{j}}{2\,\Delta x^2} - \gamma_r\,\frac{N_i^{j+1} + N_i^{j}}{2} - \gamma_{S_{\mathrm{for}\ i=1}}\,\frac{N_i^{j+1} + N_i^{j}}{2} - \gamma_R\left(\frac{N_i^{j+1} + N_i^{j}}{2}\right)^2 - \gamma_A\left(\frac{N_i^{j+1} + N_i^{j}}{2}\right)^3 + \frac{G^{j+1} + G^{j}}{2} \qquad (S1)$$

The superscript and the subscript represent the grid number of time and depth, respectively. First, we assume that $N' = N^{j+1},\ N = N^{j},\ \Delta N = N' - N$, then the radiative recombination term can be rewritten with one linear term of *N'* as follows,

$$\left(\frac{N + N'}{2}\right)^2 = \left(\frac{2N + \Delta N}{2}\right)^2 = \frac{4N^2 + 4N\cdot\Delta N + \Delta N^2}{4} \approx \frac{4N^2 + 4N\cdot\Delta N}{4} = N\cdot(N + \Delta N) = N\cdot N' \qquad (S2)$$

Similarly, the Auger term also can be rewritten with a linear term of *N'* as follows,

$$\left(\frac{N+N'}{2}\right)^3 = \left(\frac{2N+\Delta N}{2}\right)^3 = \frac{8N^3 + 12N^2\cdot\Delta N + 6N\cdot\Delta N^2 + \Delta N^3}{8}$$
$$\approx \frac{8N^3 + 12N^2\cdot\Delta N + 6N\cdot\Delta N^2}{8}$$
$$= \frac{-4N^3 - 12N^2\cdot(N+\Delta N) + 24N\cdot\frac{4N^2+4N\cdot\Delta N+\Delta N^2}{4}}{8}$$
$$= \frac{-4N^3 - 12N^2\cdot N' + 24N^2\cdot N'}{8}$$
$$= -\frac{1}{2}N^3 + \frac{3}{2}N^2\cdot N' \qquad (S3)$$

Then, substitution of Eqs. (S2) and (S3) in Eq. (S1) yields

$$\frac{N_\mathrm{i}^{j+1} - N_\mathrm{i}^{j}}{\Delta t} = D\frac{N_{i+1}^{j+1} - 2N_i^{j+1} + N_{i-1}^{j+1} + N_{i+1}^{j} - 2N_i^{j} + N_{i-1}^{j}}{2\,\Delta x^2} - \gamma_r\frac{N_i^{j+1}+N_i^{j}}{2} - \gamma_{S_{\mathrm{for}\ i=1}}\frac{N_i^{j+1}+N_i^{j}}{2} - \gamma_R\, N_i^{j}\cdot N_i^{j+1} - \gamma_A\left[-\frac{1}{2}\left(N_i^{j}\right)^3 + \frac{3}{2}\left(N_i^{j}\right)^2\cdot C_i^{j+1}\right] + \frac{G^{j+1}+G^{j}}{2} \qquad (S4)$$

II. Electron-phonon thermalization process

The scenario of carrier relaxation through hot-phonon emission was frequently used in semiconductors [2]. The photoexcited carriers relax with a characteristic time τ_e by emitting the optical phonons. Usually, only those optical phonons located within small volume (~10^{-2} in Ge, which is estimated according to the band structure and initial carrier distribution) of the Brillouin zone are involved in the hot carrier (electrons in conduction band and holes in valence band) relaxation. Thus, an optical-phonon population in large excess of the equilibrium value is established near zone center.

Finally, the optical phonons decay through the emission of longitudinal acoustic phonons near the zone boundaries with a characteristic time τ_p. The latter interaction can be considered to bring the optical phonons into equilibrium with the lattice. Quasiequilibrium distribution of all three systems is assumed to be established via collisions and multiple emission and absorption of optical phonons so that the electrons (holes), optical phonons, and lattice have time-dependent temperatures $T_e(T_h)$, T_p and T_L, respectively. In thermalization regime, the electron (hole) temperature decreases rapidly from its initial temperature; meanwhile, the optical-phonon temperature rises via electron-phonon coupling. In van Driel's studies[2], if τ_p is very small then the equilibrium time τ_e is ~5 ps, which is consistent with our observation in the case of pump fluence 135 μJ/cm^2 in Fig. S1. Furthermore, Woerner *et al.* [3] also observed the carrier relaxation with same timescale (~10 ps) via emission of optical phonons in *p*-type Ge. In summary, the relaxation of hot carriers is relatively fast compared with the spatial concentration diffusion and Auger effect, which are the main issues in this study. Thus, we neglected the relaxation processes of hot carriers while we analyzed the spatial concentration diffusion and Auger effect in Ge.

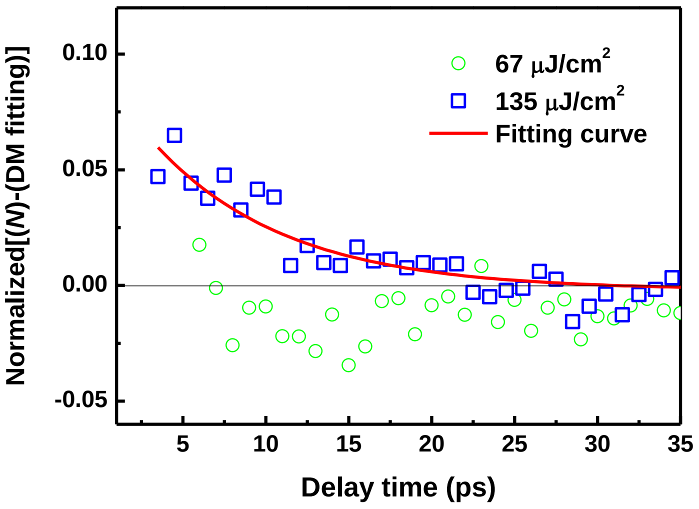

Figure S1. The difference between experimental data and the fitting of diffusion model.

Solid lines: the fitting of exponential decay function.

III. More discussion about the oscillation feature at 2000 cm^{-1}

Taking the following two points into consideration, the oscillation at 2000 cm^{-1} is not caused by interband transitions.

1. Figure S2 shows the MIR reflectance and transmittance spectra of Ge. It is obviously that the transmittance spectrum shows the band transition absorption peak at 6000 cm^{-1}, but reflection spectrum does not show the same feature. Thus, we believe that the reflectance seems unlikely to show the band absorption. Similarly, even if there has band gap energy near 2000 cm^{-1}, the transient reflectance change spectrum will not carry the feature of absorption.

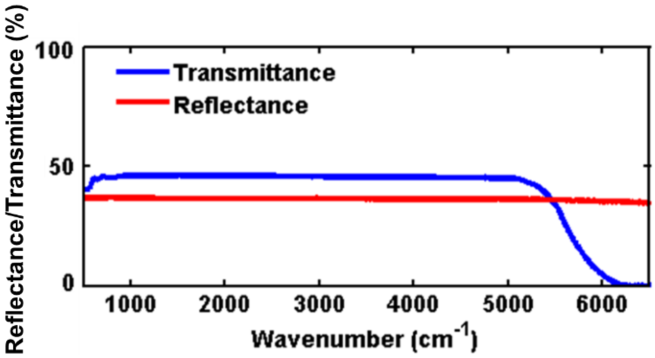

Figure S2 Reflectance (red line) and transmittance (blue line) spectra of Ge, which are measured by Fourier transform infrared spectroscopy (FTIR, Bruker, VERTEX 70/70v, incident angle 45°)

2. If we consider the intraband transition, the transition between heavy hole and light hole band is possible to happen at 2000 cm^{-1}. However, the relaxation time of intraband transitions is sub-ps at room temperature [4], which is much shorter than the relaxation time of the oscillation feature observed in our experimental data (over 30 ps as shown in Fig. 6 of manuscript).

References:

1. Crank, J. & Nicolson, P. A practical method for numerical evaluation of solutions of partial differential equations of the heat conduction type. *Proc. Camb. Phil. Soc.* **43** (1), 50-67 (1947).

2. Van Driel, H. M. Influence of hot phonons on energy relaxation of high-density carriers in germanium. *Phys. Rev. B* **19,** 5928 (1979).

3. Woerner, M., Elsaesser, T. & Kaiser, W. Inter-valence-band scattering and cooling of hot holes in *p*-type germanium studied by picosecond infrared pulses. *Phys. Rev. B* **41**, 5463 (1990).

4. Mak, G. & van Driel, H. M. Femtosecond transmission spectroscopy at the direct band edge of germanium. *Phys. Rev. B* **49**, 16817-16820 (1994).
